# Root Electrical Capacitance Can Be a Promising Plant Phenotyping Parameter in Wheat

**DOI:** 10.3390/plants11212975

**Published:** 2022-11-04

**Authors:** Imre Cseresnyés, Klára Pokovai, Judit Bányai, Péter Mikó

**Affiliations:** 1 Institute for Soil Sciences, Centre for Agricultural Research, ELKH, Herman Ottó út 15, H-1022 Budapest, Hungary; 2 Agricultural Institute, Centre for Agricultural Research, ELKH, Brunszvik u. 2, H-2462 Martonvásár, Hungary

**Keywords:** flag leaf, grain yield, in situ root methods, nutritional status, root activity

## Abstract

As root electrical capacitance (C_R_*) was assumed to depend on the stem properties, the efficiency of measuring C_R_* at flowering for whole-plant phenotyping was assessed in five wheat cultivars in three replicate plots over two years. Linear regression analysis was used to correlate C_R_* with plant-size parameters and flag-leaf traits (extension and SPAD chlorophyll content) at flowering, and with yield components at maturity. The plot-mean C_R_* was correlated with the plot leaf area index (LAI), the chlorophyll quantity (LAI×SPAD), and the grain yield across years. At plant scale, C_R_* was found to show the strongest positive regression with total chlorophyll in the flag leaf (flag leaf area × SPAD; R^2^: 0.65–0.74) and with grain mass (R^2^: 0.55–0.70) for each cultivar and year (*p* < 0.001). Likewise, at plot scale, the regression was strongest between C_R_* and the LAI×SPAD value (R^2^: 0.86–0.99; *p* < 0.01) for the cultivars. Consequently, C_R_* indicated the total plant nutrient and photosynthate supply at flowering, which depended on root uptake capacity, and strongly influenced the final yield. Our results suggested that the polarization of the active root membrane surfaces was the main contributor to C_R_*, and that the measurement could be suitable for evaluating root size and functional intensity. In conclusion, the capacitance method can be applied for nondestructive whole-plant phenotyping, with potential to estimate root and shoot traits linked to the nutrient supply, and to predict grain yield. C_R_* can be incorporated into allometric models of cereal development, contributing to optimal crop management and genetic improvement.

## 1. Introduction

An extensive root system is critical for improved biomass production, crop grain yield (GY), and yield stability when water and/or nutrient availability is limited [1]. Although the significance of root phenotyping in plant breeding has been widely recognized [2], root studies often lag behind those of aboveground organs due to methodological constraints [3]. Destructive root investigations and soil disturbance should usually be avoided during the growing season (particularly in small-plot experiments), and the extraction of the intact root system from the field soil is not feasible [4]. Therefore, rapid, cost-effective in situ screening methods deserve particular attention.

Initially, Chloupek [5] observed a significant correlation between root system size (RSS) and root electrical capacitance (C_R_), measured between a ground electrode inserted into the soil and a plant electrode fixed on the stem, using a low-frequency (1 kHz) alternating current (AC). The first conceptual model for C_R_ measurement [6] considered roots to be equivalent to leaky cylindrical capacitors, in which the membrane dielectrics store electric charges, separating the conductive root sap and soil solution, which represent the two capacitor plates. Polarization induces changes in the amplitude and phase of the AC signal, forming an electrical capacitance that is proportional to the interfacial membrane surface area. Several experiments verified that a large part of the root system played a role in determining the capacitance response [7,8]. Conversely, others questioned the efficiency of the capacitance method, showing that distal roots conduct only a small fraction of the current due to localized charge leakage in the most proximal root segments [9,10]. According to Dietrich et al. [11], the capacitance chiefly derives from the tissues between the substrate surface and the plant electrode, and depends on the stem cross-sectional area (CSA). Thus, the correlation between C_R_ and RSS is merely due to the allometric relationship between the above- and belowground plant traits. A root excision experiment in potted maize partially resolved the conflicting findings, demonstrating that C_R_ was mainly determined by the roots in the soil, but was also influenced by the size-dependent capacitance of the stem base (which was responsible for 31–39% of the total capacitance) [12]. Furthermore, roots make the greatest contribution to the capacitance when the surrounding soil is dry and, therefore, less electrically conductive than the roots [13]. A generally accepted advantage is that, as C_R_ depends not only on root size but also on root tissue characteristics (e.g., maturation and suberization), the method provides related information on root functionality [6,8,10,14].

Numerous studies confirmed the usability of the capacitance technique for assessing RSS in hydroponics and potted soils (reviewed by Ehosioke et al. [15]), and later for field-grown crops [16,17]. Importantly, C_R_ data are only comparable when the same species is grown in the same soil type with equal soil water content (SWC), and are measured using the same electrode sizes in identical positions [18,19]. However, the marked effect of SWC on C_R_ can be calculated using a species-specific experimental function, which allows the measured C_R_ to be converted into a saturation capacitance (C_R_*; which can be measured in water-saturated soil) on the basis of the SWC detected in the root zone of the same plant [14]. The calculation of C_R_* allows the comparison of capacitance data collected under spatially or temporally variable SWC, which is particularly relevant for field studies.

The capacitance method has been considered as a simple, high-throughput root phenotyping tool for evaluating a large number of individual plants repeatedly during their life cycle [18]. Moreover, the selected plants can be harvested at maturity to assess seed quality, to measure grain mass (GM), and to use the seeds for further breeding work [17]. Significant positive correlations between the C_R_-based RSS and GM were shown for field-grown barley [16,18,20], wheat [21,22], and canola [23]. Cereals are reported to show a strong seasonal pattern in root production, with maximum root size and uptake activity during flowering, in parallel with the peaks of leaf area and total plant transpiration [24,25]. It was demonstrated earlier that the CR* of winter wheat cultivars peaked at anthesis, and this date proved to be a better predictor of GM under different cultivation conditions than the direct measurement of C_R_ [26,27].

As discussed previously, root capacitance is affected by the size and histological characteristics of the stem, and, thus, indirectly by other aboveground plant properties [10,11,12]. Significant correlations between root and shoot traits and the yield components are well documented for cereal crops including wheat [3]. A deep understanding of these stable allometric relationships can be particularly beneficial for estimating plant variables that are problematic to measure, such as RSS traits, and for predicting growth parameters and GY [28]. In wheat, more vigorous root growth and soil resource acquisition are closely linked to increased leaf area formation, enhanced carbohydrate and N accumulation in the vegetative organs until flowering, and the subsequent translocation to the grains during maturity [2]. As the wheat flag leaf produces nearly half the photosynthates required for grain filling, its size, morphology, and chlorophyll content (e.g., in SPAD unit) at the flowering stage are powerful indicators of plant nutritional status and are important determinants of yield-related traits [29,30].

While the measured capacitance was assumed to depend on the stem properties, to date, there have been no reports demonstrating the suitability of the method for rapid, whole-plant phenotyping in situ in the field. To fill this knowledge gap, the present study was planned to perform capacitance measurements to evaluate shoot parameters related to aboveground production and nutrient conditions, and to predict various yield components in a two-year study involving five winter wheat cultivars in three replicate plots. Specifically, correlations were determined between C_R_* measured on individual plants at anthesis with: (1) aboveground parameters at the same phenophase, such as plant height (PH), CSA, PH×CSA value (used to replace shoot biomass), spike length (SL), flag leaf length (FLL), flag leaf width (FLW), flag leaf area (FLA), flag leaf chlorophyll content (SPAD), and FLA×SPAD derivative (representing the total quantity of chlorophyll in the flag leaf); (2) parameters measured after harvest at physiological maturity, such as total aboveground biomass (TAB), GM, and grain number (GN). Moreover, plot-scale investigations were carried out on the two-year data pool: the plot average of C_R_* was correlated with the ceptometer-based leaf area index (LAI) detected at anthesis, with the LAI×SPAD variable, considered as an indicator of crop N status [31], and with the plot GY obtained at the terminal harvest. The study was designed to preclude both soil disturbance and any destructive plant investigations and sample collection before full maturity, and furthermore to avoid time-consuming and costly laboratory analyses.

## 2. Results

### 2.1. Plant and Plot-Scale Parameters

**Figure 1:**
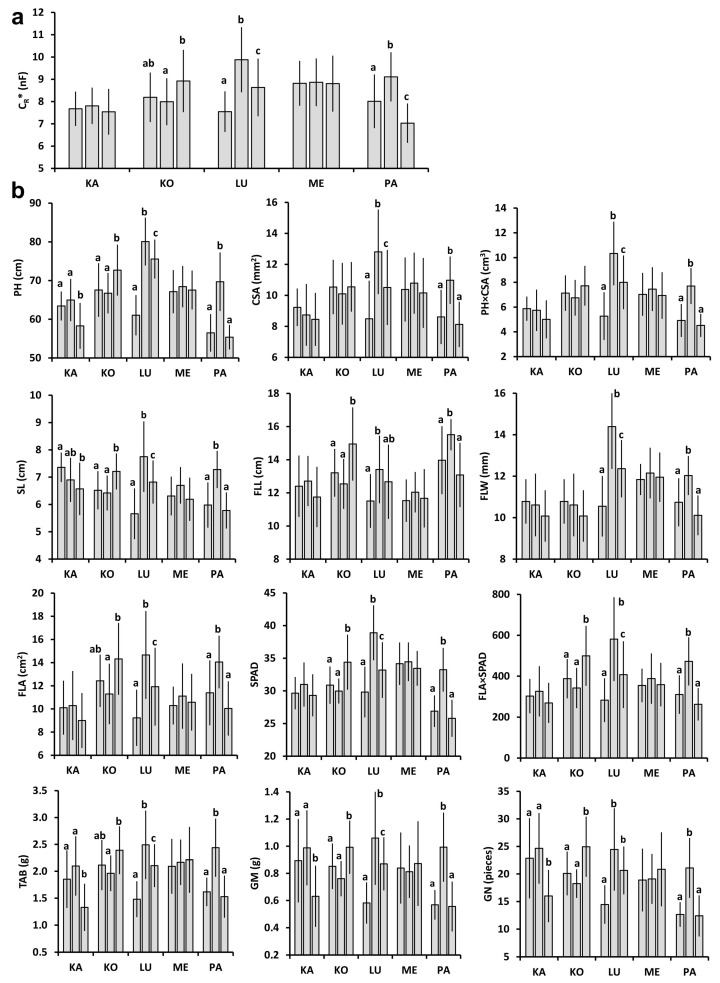
Measured plant parameters (mean ± SD; n = 20) for the five wheat cultivars (KA: Káplár; KO: Kolo; LU: Lucilla; ME: Ménrót; PA: Pántlika) in three replicate plots (A, B, and C from left to right) in 2021. Different lower-case letters indicate significant differences (ANOVA with Tukey’s post hoc test) between the three plots within a cultivar. (**a**) C_R_*: saturation root electrical capacitance; (**b**) PH: plant height; CSA: stem cross-sectional area; SL: spike length; FLL: flag leaf length; FLW: flag leaf width; FLA: flag leaf area; SPAD: chlorophyll content in flag leaf; TAB: total aboveground biomass; GM: grain mass; GN: grain number.

**Figure 2:**
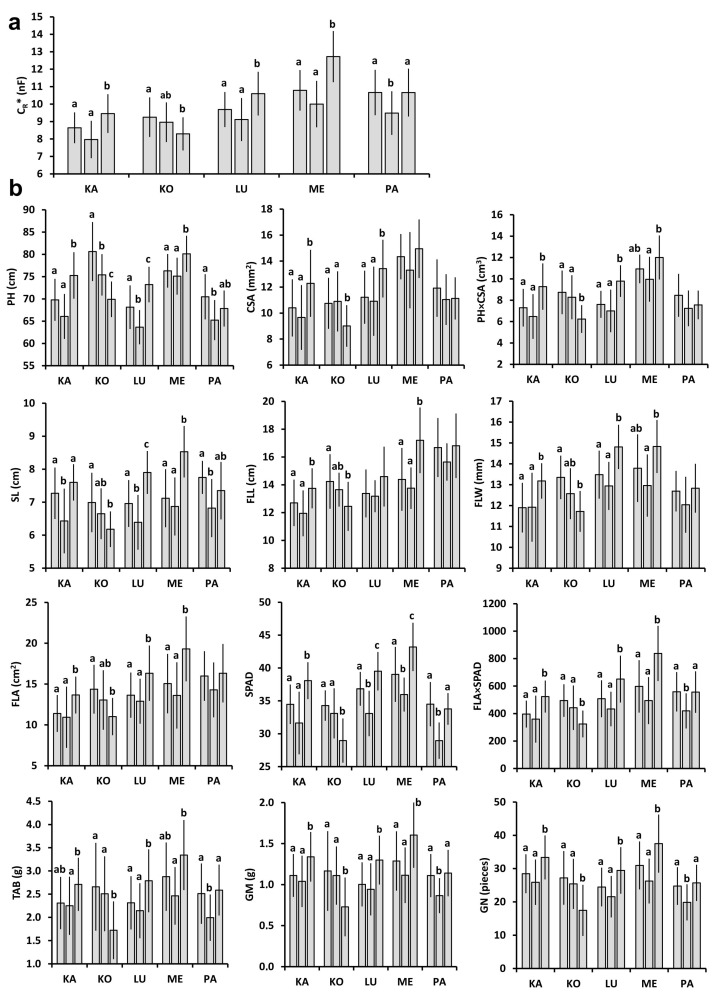
Measured plant parameters (mean ± SD; n = 20) for the five wheat cultivars (KA: Káplár; KO: Kolo; LU: Lucilla; ME: Ménrót; PA: Pántlika) in three replicate plots (A, B, and C from left to right) in 2022. Different lower-case letters indicate significant differences (ANOVA with Tukey’s post hoc test) between the three plots within a cultivar. (**a**) C_R_*: saturation root electrical capacitance; (**b**) PH: plant height; CSA: stem cross-sectional area; SL: spike length; FLL: flag leaf length; FLW: flag leaf width; FLA: flag leaf area; SPAD: chlorophyll content in flag leaf; TAB: total aboveground biomass; GM: grain mass; GN: grain number.

The mean C_R_* in the plots (n = 20) ranged from 7.03 to 9.88 nF in 2021 (Figure 1a) and from 7.97 to 12.72 nF in 2022 (Figure 2a) for all the wheat cultivars tested (θ_rel_, from which C_R_* was calculated, was between 0.37 and 0.42 in 2021, and between 0.18 and 0.22 in 2022 at anthesis). Except for Káplár and Ménrót in 2021, significant differences in C_R_* were found between the three replicate plots for each cultivar and year. All five cultivars had a higher mean C_R_* (n = 60) in 2022 than in 2021; the relative change was only 6% for Kolo, 13% for Káplár and Lucilla, and highest for Ménrót (27%) and Pántlika (28%). The observed variability in C_R_* was in accordance with the 12 individual plant parameters measured or calculated for each cultivar and year (Figure 1b and Figure 2b). However, PH, SL, and the post-harvest parameters (TAB, GM, and GN) for Káplár proved to be significantly lower in plot A than in B and C. Furthermore, no statistical difference was found in CSA, PH×CSA, and flag-leaf extension (FLL, FLW, and FLA) between the plots for Pántlika in 2022. The mean values of the individual plant parameters were usually higher in 2022 than in 2021 (except for PH for Lucilla, and CSA and SL for Kolo); the relative change was smallest for Kolo (−2–20%), followed by Lucilla (−5–29%), Káplár (2–42%), Pántlika (12–52%), and Ménrót (14–75%).

**Figure 3:**
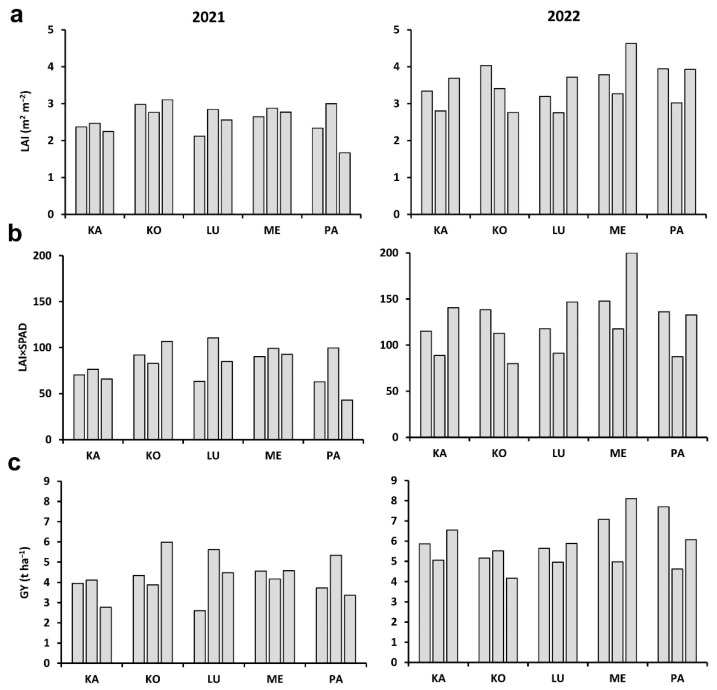
(**a**) Leaf area index (LAI), (**b**) LAI×SPAD (chlorophyll amount in flag leaf) value, and (**c**) grain yield (GY) for the five wheat cultivars (KA: Káplár; KO: Kolo; LU: Lucilla; ME: Ménrót; PA: Pántlika) in three replicate plots (A, B, and C from left to right) in two years.

The plot LAI values ranged from 1.67 to 3.11 m^2^ m^−2^ in 2021, and from 2.76 to 4.73 m^2^ m^−2^ in 2022 (Figure 3a). The mean LAI (n = 3) was higher in 2022 than 2021 for each cultivar, by 15% for Kolo, 29% for Lucilla, 39% for Káplár, 41% for Ménrót, and 55% for Pántlika. The LAI×SPAD values were between 43 and 111 in 2021 and between 80 and 200 in 2022 (Figure 3b); the relative increase in the second year was 18%, 38%, 62%, 65%, and 73% for Kolo, Lucilla, Káplár, Ménrót, and Pántlika, respectively. Due to the organic conditions, the plots produced variable and relatively low GY, ranging from 2.61 to 5.99 t ha^−1^ in 2021 and from 4.17 to 8.11 t ha^−1^ in 2022 (Figure 3c). GY was higher in 2022 than 2021, by 5%, 30%, 48%, 53%, and 61% for Kolo, Lucilla, Pántlika, Ménrót, and Káplár, respectively. The order established for each plot-scale parameter for a given cultivar in the three replicated plots corresponded with the means of the plant parameters measured, except for the GY of Kolo, which was somewhat higher in plot B than plot A in 2022.

### 2.2. Relationships between Individual Plant Parameters

The pairwise relationships between all 13 individual plant parameters were evaluated by linear regression. The R^2^ values and significance levels (based on the F test) are given in the Supplementary materials (Tables S1–S5). Here, however, only the relationships between C_R_* and other plant parameters are considered, the regression line slopes and *y*-intercepts of which are summarized in Table S6.

**Figure 4:**
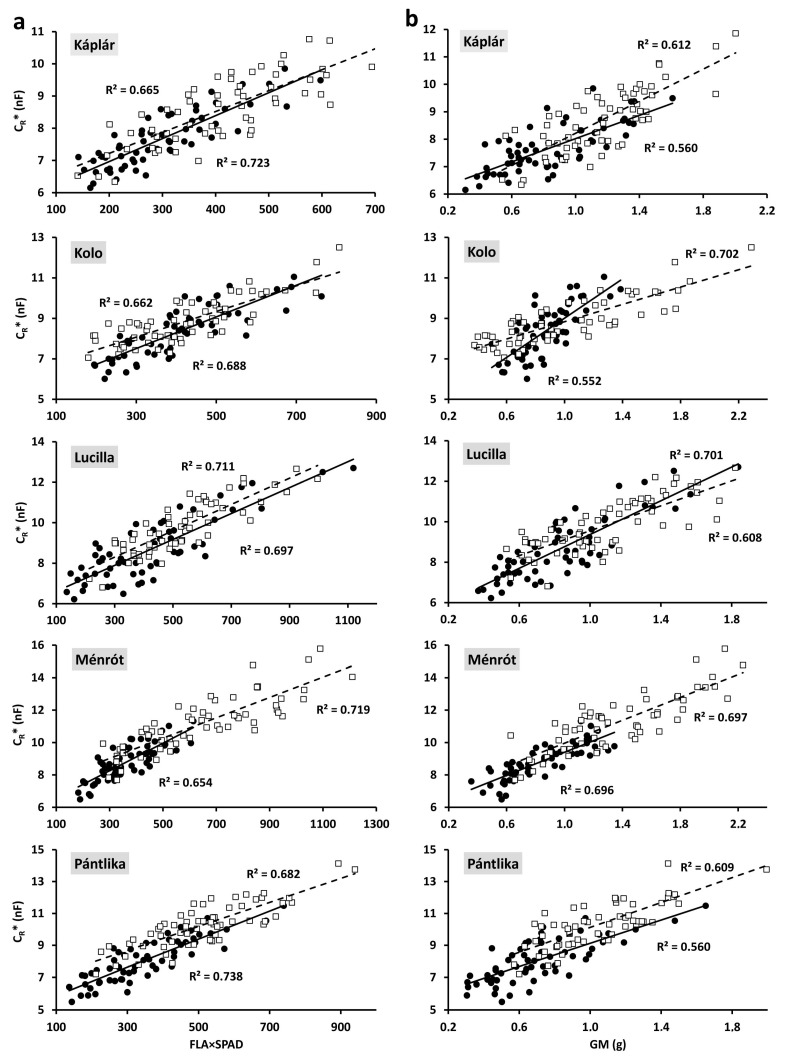
Relationship between the saturation root electrical capacitance (C_R_*) and (**a**) FLA (flag leaf area) × SPAD value (chlorophyll content in flag leaf) and (**b**) grain mass (GM) for the five wheat cultivars in 2021 (●; solid line) and 2022 (□; dashed line). All the regressions were significant at *p* < 0.001 (n = 60).

All the regressions were highly significant (*p* < 0.01 or 0.001), apart from C_R_*–PH for Kolo in 2022 (*p* = 0.11; Table 1). C_R_* exhibited the strongest linear correlations with FLA (R^2^: 0.564–0.723), FLA×SPAD in most cases (R^2^: 0.654–0.738; Figure 4a), and GM (R^2^: 0.552–0.752; Figure 4b). The relationship was somewhat weaker between C_R_* and FLL (R^2^: 0.474–0.680), FLW (R^2^: 0.507–0.664), SPAD (R^2^: 0.328–0.628), TAB (R^2^: 0.522–0.645), and GN (R^2^: 0.489–0.696). Relatively, the poorest correlations were obtained between C_R_* and aboveground plant size parameters, such as PH (R^2^: 0.043–0.412), CSA (R^2^: 0.129–0.533), PH×CSA (R^2^: 0.173–0.533), and SL (R^2^: 0.262–0.588). Similar regression fits were found in the two years for the relationship between C_R_* and the flag-leaf and post-harvest parameters. Nevertheless, the correlations between C_R_* and plant size parameters were usually weaker in 2022.

The results of ANOVA (Table 2) showed that the regression line slopes were significantly affected by the wheat cultivar (CV; except for the C_R_*–SL and C_R_*–FLL relationships), but not by the year (Y; except for the C_R_*–GM relationship). The CV×Y interaction had a significant effect on the line slope for the regressions between C_R_* and the PH×CSA and post-harvest parameters. For each plant parameter, highly significant differences between cultivars and years were found for the *y*-intercept of the regression line, and in most cases, a significant CV×Y interaction effect was also detected. The line intercepts usually had higher values in 2022 than in 2021 (Table S6).

### 2.3. Correlations between Plot-Scale Parameters

**Figure 5:**
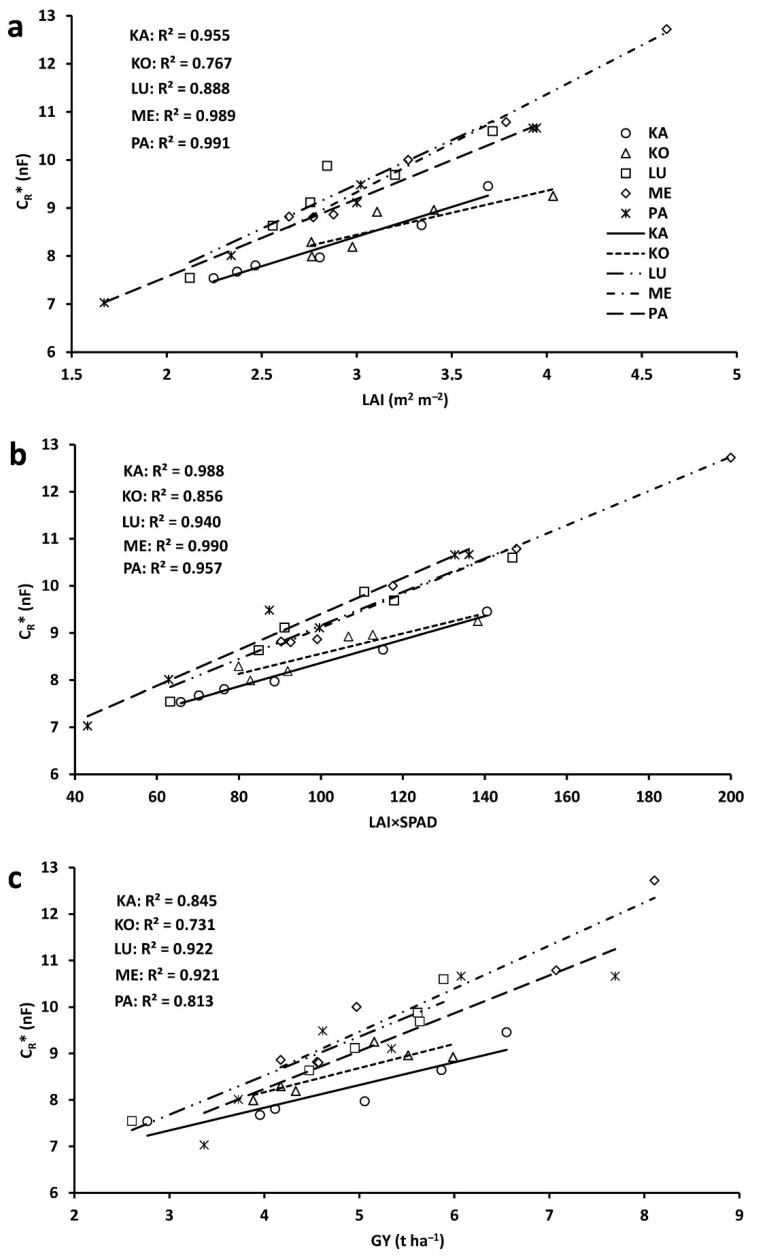
Relationship between the saturation root electrical capacitance (C_R_*; means of 20 plants from a plot) and (**a**) leaf area index (LAI), (**b**) LAI×SPAD value (chlorophyll quantity in flag leaf), and (**c**) grain yield (GY) for the five wheat cultivars (KA: Káplár; KO: Kolo; LU: Lucilla; ME: Ménrót; PA: Pántlika) grown in three plots. For each cultivar, data were pooled across the two years. All the regressions were significant at *p* < 0.05 (n = 6).

When pooling the data across years (n = 6), the plot-mean C_R_* proved to be significantly correlated with plot LAI (R^2^: 0.767–0.991; *p* < 0.05; Figure 5a), LAI×SPAD (R^2^: 0.856–0.990; *p* < 0.01; Figure 5b), and GY (R^2^: 0.731–0.922; *p* < 0.05; Figure 5c) for each cultivar. ANOVA showed significant differences between the cultivars in the slope and *y*-intercept of the regression lines for the C_R_*–LAI and C_R_*–LAI×SPAD relationships, and in the y-intercept for C_R_*–GY. The overall regressions, obtained by pooling the data across years and cultivars (n = 30), were also found to be highly significant (*p* < 0.001), with R^2^ values of 0.774, 0.855, and 0.758 for C_R_*–LAI, C_R_*–LAI×SPAD, and C_R_*–GY relationships, respectively.

## 3. Discussion

### 3.1. Linear Regression

In most cases, positive linear correlations were obtained at a high significance level between C_R_* and plant-size parameters (PH, CSA, PH×CSA, and SL), flag-leaf traits (FLL, FLW, FLA, SPAD, and FLA×SPAD), and post-harvest (yield) parameters (TAB, GM, and GN) for each wheat cultivar and year. Previous studies reported comparable relationships (obtained using various correlation methods) between wheat root biomass or root length and shoot size parameters, but usually revealed weaker correlations between root size and GM [2,3,32,33]. However, these studies were based on tube or rhizobox experiments, or were carried out in the field, but used destructive (soil core) methods for root investigations. Furthermore, Postic et al. [33] obtained an R^2^ of 0.48 for linear regression between minirhizotron-based root length density and GY for field-grown wheat.

Linear regressions between C_R_ and GM generally provided R^2^ values from 0.21 to 0.42 for cereals grown in a dry environment, whereas no significant correlations (R^2^: 0.11–0.14) were found under optimal water conditions [16,20,21,22]. In the present work, much closer C_R_*–GM correlations were usually obtained not just in 2021 (R^2^: 0.55–0.70), but also in 2022 (R^2^: 0.61–0.70), when no considerable drought prevailed. This was likely due to the application of C_R_* instead of directly measured C_R_ values, which mitigated the effect of SWC variability on the capacitance data.

The cultivar, year, and their interaction were also shown to influence the regression equation parameters. This is in line with previous reports that allometric relationships in wheat depended on the genotype, cultivation technique, and environmental conditions [34,35]. Moreover, the differences in the mean C_R_* between the dry 2021 and the more favorable 2022 years reflected the responsiveness of the cultivar to variable weather conditions, i.e., the high tolerance of Kolo and the sensitivity of Ménrót and Pántlika.

### 3.2. Indication of Root Functions

A notable finding presented here is that C_R_* was correlated to the greatest extent with flag-leaf traits, particularly with the total amount of chlorophyll (FLA×SPAD), and with GM for each cultivar and year. Thus, C_R_* served as an indicator of the total nutrient (mainly N) uptake and photosynthate supply of the plant at the flowering stage. The reason for this could be that plant nourishment is chiefly determined by the extension and uptake capacity of the root system, especially when the plant faces drought or suboptimal nutrient supplies (i.e., organic farming in the present case). As the accumulated resources are transferred to the grains during the maturity stages, and, together with environmental conditions, have a decisive effect on GM [31,36], C_R_* provided a reasonable estimate of the yield parameters as well.

These results strongly suggest that C_R_* depends, at least partially, on the polarization of root membranes. Consequently, capacitance measurement can be considered to be a valid method for assessing root size and functional intensity in the field at the flowering stage, when plants exhibit maximum physiological activity and resource demand during their ontogeny [1]. This contradicts the findings of Dietrich et al. [11], who stated that the roots had a negligible role in the capacitance response, which was governed by stem-base polarization, and correlated linearly with CSA (R^2^: 0.77–0.81 for barley). The present work revealed much poorer C_R_*–CSA correlations in 2021 (R^2^: 0.36–0.53) and particularly in 2022 (R^2^: 0.13–0.46). Furthermore, these fits were weaker for each cultivar and year than for the regressions between C_R_* and flag-leaf parameters (R^2^: 0.33–0.74) or GM (R^2^: 0.55–0.70), providing further evidence of the influence of the root system on the measured capacitance. This confirms previous reports that AC is able to penetrate to deeper roots, particularly when the soil is much drier than the field capacity and the roots offer a more favorable current path [13,37]. It should be noted that though C_R_* values are associated with water-saturated soil, the capacitance measurements in the present study were always taken in considerably drier soil (θ_rel_ from 0.18 to 0.42 vs. θ_rel_ of 0.65 at field capacity). In agreement with observations by Dietrich et al. [11], a previous study verified the role of the stem in the electrical circuit and, thus, in the C_R_ values detected [12]. This is likely the reason why higher *y*-intercept values were obtained for the regressions between C_R_* and individual plant parameters in 2022, when the wheat cultivars had greater aboveground production and consequently higher CSA due to the more favorable rainfall conditions.

At plot scale, crop performance was characterized nondestructively with LAI and the LAI×SPAD derivative at anthesis, and with GY at maturity. These parameters were significantly correlated with the plot-mean of C_R_* (R^2^: 0.73–0.99), although the data were pooled across years for the analysis. The LAI×SPAD value showed the strongest correlation with C_R_* for all the cultivars, except Pántlika, for which the LAI–C_R_* regression had the best fit. This is concurrent with the plant-scale regression analysis, as C_R_* indicates the plant nutritional status linked to root functional activity. Similar results were obtained when all five cultivars were pooled for data analysis. This suggests that the capacitance method provides information on crop development at the population level. However, further research targeted at data collection over more than two years is undoubtedly needed to strengthen the present findings.

## 4. Materials and Methods

### 4.1. Study Site and Wheat Cultivation

**Figure 6:**
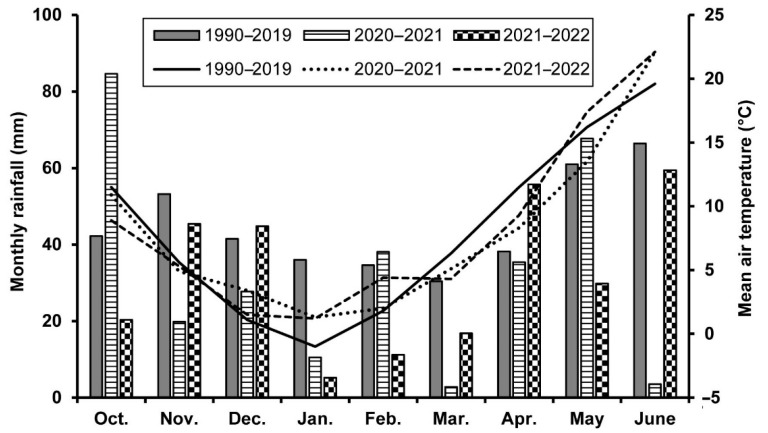
Monthly rainfall (columns) and mean air temperature (lines) at the experimental site of the Centre for Agricultural Research (Martonvásár, Hungary) during the winter wheat growing season. Long-term (1990–2019) average is displayed for reference.

The field study was conducted in the 2020–2021 and 2021–2022 winter wheat growing seasons on the organic experimental area of the Centre for Agricultural Research, Martonvásár, Hungary (47°18′ N, 18°46′ E, 108 m asl.). The soil is classified as Haplic chernozem (36% sand, 41% silt, and 23% clay) according to the FAO-WRB system [38], with a pH of 7.69, 1.75% CaCO_3_, 3.89% humus, 2015/401/485 mg kg^−1^ total N/P/K, 1.41 g cm^−3^ bulk density, and 0.314 and 0.482 cm^3^ cm^−3^ field capacity and saturation water content, respectively. The meteorological data were collected by an on-site weather station. The area has a continental climate with a mean (1990–2019) annual temperature of 11.0 °C (−1.0 °C in January and 21.3 °C in July) and annual precipitation of 556 mm, with 196 mm during the main winter wheat growing season, from March to June (Figure 6). In 2021, April and May were very cold (3 °C below the long-term mean), and March and June were extremely dry with only 3 and 4 mm of total rainfall, respectively. More favorable growing conditions with no considerable drought prevailed in 2022. Although only half of the mean monthly precipitation fell in May, rainfall was evenly distributed over the month, mitigating the drought stress.

Five winter wheat (*Triticum aestivum* L.) cultivars, namely (Mv) Káplár, (Mv) Kolo, (Mv) Lucilla, (Mv) Ménrót, and (Mv) Pántlika, were machine-sown in late October 2020 and 2021 at a rate of 500 seeds m^−2^. The plots were 6 m long and 1 m wide, containing 8 rows spaced 12.5 cm apart, and were separated by a 0.5 m border. Three replicate plots (A, B, and C) were arranged randomly in slightly different places each year to ensure crop rotation on the experimental area. Mineral fertilizers and artificial chemicals were not used, in compliance with organic farming.

### 4.2. Electrical Capacitance Measurement

At the anthesis stage (BBCH 65; in mid to late May, depending on the cultivar and year), 20 plants were randomly selected from the central rows of each plot to avoid the border effect, and were individually tagged. SWC was measured in the 0–12 cm layer at a distance of 5 cm from each plant using a calibrated CS620 handheld TDR meter (Campbell Sci. Ltd., Loughborough, UK). The volumetric SWC value was divided by the saturation water content to obtain the relative water saturation of the soil (θ_rel_). Thereafter, C_R_ (modeled by a parallel equivalent circuit) was measured for the sample plants with a U1733C portable LCR instrument (Agilent Co., Ltd., Penang, Malaysia) at 1 kHz, 1 V AC. The ground electrode was a sharp stainless-steel rod, 15 cm in length and 6 mm in diameter (303S31; RS Pro GmbH., Gmünd, Austria), inserted vertically into the soil to a depth of 12 cm and 5 cm away from the plant (in the same position and depth as the TDR probe). The plant electrode was clamped to all the basal parts of the plant 15 mm above ground level [20], after smearing them with conductivity gel. Saturation capacitance was calculated from all the C_R_–θ_rel_ data pairs using the previously parameterized exponential function C_R_* = C_R_ × 5.807 × e^−1.775θrel^, which was (see Cseresnyés et al. [14] for details) to ensure data comparability.

### 4.3. Measurement of Individual Plant and Plot-Scale Parameters

Immediately after the electrical measurements, PH and SL were measured with a steel tape (±1 mm) from the soil surface and from the base of the spike, respectively, to the tip of the spike excluding the awns [28]. The stem diameter (SDI) was recorded in the middle of the second internode [39] using a digital caliper (±0.1 mm), and CSA = [(SDI/2)^2^ × π] was calculated. FLL was measured from the base to the tip of the flag leaf with a steel tape (±1 mm). The FLW measurement was taken at the widest part of the flag leaf with a digital caliper (±0.1 mm), and FLA was defined as FLL × FLW × 0.75 [30]. The chlorophyll content of the flag leaf was detected in situ with a handheld SPAD-502 meter (Konica Minolta Inc., Osaka, Japan) during the midday hours. Three SPAD readings, taken in the middle third of the adaxial (top) side of the blade avoiding the edges, were averaged for each leaf [29]. LAI was detected nondestructively in the central part of the plots between 11 a.m. and 1 p.m. with an Accupar LP-80 ceptometer (Meter Group Inc., Pullmann, WA, USA). The 80 cm long sensor was placed parallel and perpendicular to the wheat rows (representing a 0.8 m × 0.8 m area), and was read twice in each position to calculate the plot LAI as a mean of 22 readings [40]. The PH×CSA and FLA×SPAD values were determined for each plant by multiplying the corresponding plant parameters. The LAI×SPAD derivative was calculated on the basis of plot LAI and the mean SPAD obtained from 20 plants grown in the same plot.

At wheat maturity (in early July), the tagged plants were cut at ground level, put in separate paper bags, and dried at 70 °C to constant weight to determine TAB (±0.001 g). The spikes were hand-threshed, GN was counted, and GM per plant was weighed. The plots were then harvested and threshed by machine, and GY was determined as t ha^−1^ for each plot.

### 4.4. Data Analysis

The data were analyzed in R programming language [41]. One-way ANOVA with Tukey’s post hoc test was performed to compare the means of plant parameters between the three replicate plots (a statistical comparison of the two years was not aimed at). Normality and the equality of variances in the data groups were proved with the Shapiro–Wilk test and Levene test, respectively. Statistical significance was assessed at *p* < 0.05. For each wheat cultivar, a linear regression model was used to describe the relationship between (i) the parameters measured for the individual plants (n = 60), and (ii) the plot-scale parameters by pooling the data across the years (n = 6). The coefficient of determination (R^2^) was used to assess the fit of the regressions, and the F test was performed to determine the significance level. ANOVA was used to evaluate the effect of cultivar (CV), year (Y), and the CV×Y interaction on the line slopes and *y*-intercepts.

## 5. Conclusions

C_R_* was shown to be an effective parameter to indirectly measure the functional size and uptake capacity of the root system. Thus, it has potential to estimate the aboveground plant traits, particularly those that reflect the nutrient supply at flowering, and, in turn, to predict grain yield for a given cultivar. The nondestructive, high-throughput, and cheap capacitance method could be useful for optimizing the phenotype characteristics, not only of the root but also of the shoot, which are decisive for final yield. Although it is still far from being a routine technique, the present approach is capable of complementing the widely used conventional soil-intrusive and/or plant-destructive root investigations.

The inclusion of root traits (i.e., water and nutrient uptake efficiency) and flag-leaf characteristics in crop selection is fundamental for achieving progress in breeding. C_R_* can be considered as a new predictor variable in allometric models aimed at forecasting cereal growth and development. Root capacitance measurements in the field could potentially be a nondestructive tool for the optimal management and genetic improvement of crop plants.

## Figures and Tables

**Table 1:** Coefficient of determination (R^2^) values and significance levels (based on the F test) for linear regressions between the saturation root electrical capacitance (C_R_*) and various plant parameters investigated in five wheat cultivars in two years. The regressions were established for the individual plants (n = 60). The highest R^2^ for a given cultivar and year is written in bold. For abbreviations, see Figure 2. ** *p* < 0.01; *** *p* < 0.001; NS not significant.

Year	Parameter	Cultivar				
		Káplár	Kolo	Lucilla	Ménrót	Pántlika
**2021**	PH	0.281 ***	0.275 ***	0.412 ***	0.165 **	0.361 ***
	CSA	0.497 ***	0.368 ***	0.405 ***	0.355 ***	0.533 ***
	PH×CSA	0.501 ***	0.511 ***	0.475 ***	0.354 ***	0.533 ***
	SL	0.564 ***	0.338 ***	0.451 ***	0.385 ***	0.572 ***
	FLL	0.594 ***	0.680 ***	0.522 ***	0.533 ***	0.600 ***
	FLW	0.621 ***	0.507 ***	0.650 ***	0.595 ***	0.664 ***
	FLA	0.693 ***	**0.723** ***	0.680 ***	0.604 ***	0.688 ***
	SPAD	0.502 ***	0.424 ***	0.565 ***	0.533 ***	0.626 ***
	FLA×SPAD	**0.723** ***	0.688 ***	0.697 ***	0.654 ***	**0.738** ***
	TAB	0.524 ***	0.545 ***	0.645 ***	0.633 ***	0.551 ***
	GM	0.560 ***	0.552 ***	**0.701** ***	**0.696** ***	0.560 ***
	GN	0.489 ***	0.508 ***	0.662 ***	0.628 ***	0.548 ***
2022	PH	0.249 ***	0.043 ^NS^	0.149 **	0.155 **	0.268 ***
	CSA	0.464 ***	0.216 ***	0.193 ***	0.291 ***	0.129 **
	PH×CSA	0.474 ***	0.173 ***	0.255 ***	0.402 ***	0.225 ***
	SL	0.458 ***	0.262 ***	0.588 ***	0.415 ***	0.381 ***
	FLL	0.474 ***	0.602 ***	0.611 ***	0.621 ***	0.484 ***
	FLW	0.552 ***	0.581 ***	0.558 ***	0.507 ***	0.634 ***
	FLA	0.564 ***	0.681 ***	0.660 ***	0.625 ***	0.601 ***
	SPAD	0.628 ***	0.328 ***	0.605 ***	0.599 ***	0.509 ***
	FLA×SPAD	**0.665** ***	0.662 ***	**0.711** ***	**0.719** ***	**0.682** ***
	TAB	0.522 ***	0.583 ***	0.565**	0.620 ***	0.557 ***
	GM	0.612 ***	**0.702** ***	0.608 ***	0.697 ***	0.609 ***
	GN	0.552 ***	0.696 ***	0.576 ***	0.696 ***	0.603 ***

**Table 2:** ANOVA results for the effect of cultivar (CV), year (Y), and CV×Y interaction on the slope and *y*-intercept of the linear regressions established between the saturation root electrical capacitance (C_R_*) and various plant parameters. For abbreviations, see Figure 1. * *p* < 0.05; ** *p* < 0.01; *** *p* < 0.001; NS not significant.

Parameter	Slope			*y*-Intercept		
	CV	Y	CV×Y	CV	Y	CV×Y
PH	***	NS	NS	***	***	***
CSA	*	NS	NS	***	***	**
PH×CSA	**	NS	**	***	***	***
SL	NS	NS	NS	***	***	NS
FLL	NS	NS	NS	***	***	*
FLW	***	NS	NS	***	***	**
FLA	*	NS	NS	***	***	*
SPAD	***	NS	NS	***	***	***
FLA×SPAD	***	NS	NS	***	***	**
TAB	***	NS	**	***	***	***
GM	***	*	***	***	***	**
GN	***	NS	**	***	***	NS

## Data Availability

The data that support the findings of this study are available from the corresponding author upon reasonable request.

## Supplementary Materials

The following supporting information can be downloaded at: https://www.mdpi.com/article/10.3390/plants11212975/s1, Table S1: The coefficient of determination (R^2^) values and statistical significances (based on the F test) for the linear regressions between the measured individual plant parameters for wheat cultivar Káplár in two years. The regressions were established for individual plants (n = 60). C_R_*: saturation root electrical capacitance; PH: plant height; CSA: stem cross-sectional area; SL: spike length; FLL: flag leaf length; FLW: flag leaf width; FLA: flag leaf area; SPAD: chlorophyll content in flag leaf; TAB: total aboveground biomass; GM: grain mass; GN: grain number. * *p* < 0.05; ** *p* < 0.01; *** *p* < 0.001; NS not significant; Table S2: The coefficient of determination (R^2^) values and statistical significances (based on the F test) for the linear regressions between the measured individual plant parameters for wheat cultivar Kolo in two years. The regressions were established for individual plants (n = 60). For the abbreviations and symbols, see Table S1; Table S3: The coefficient of determination (R^2^) values and statistical significances (based on the F test) for the linear regressions between the measured individual plant parameters for wheat cultivar Lucilla in two years. The regressions were established for individual plants (n = 60). For the abbreviations and symbols, see Table S1; Table S4: The coefficient of determination (R^2^) values and statistical significances (based on the F test) for the linear regressions between the measured individual plant parameters for wheat cultivar Ménrót in two years. The regressions were established for individual plants (n = 60). For the abbreviations and symbols, see Table S1; Table S5: The coefficient of determination (R^2^) values and statistical significances (based on the F test) for the linear regressions between the measured individual plant parameters for wheat cultivar Pántlika in two years. The regressions were established for individual plants (n = 60). For the abbreviations and symbols, see Table S1; Table S6: The slope (±SE) and y-intercept (±SE) for the linear regressions between the saturation root electrical capacitance (C_R_*) and various plant parameters investigated in five wheat cultivars in two years. The regressions were established for individual plants (n = 60). For the abbreviations, see Table S1.
