# Supplementary material for: Root Electrical Capacitance Can Be a Promising Plant Phenotyping Parameter in Wheat
Source: Plants (Basel). 2022 Nov 4;11(21):2975. doi: 10.3390/plants11212975 (PMC9657365; doi:10.3390/plants11212975)
Supplement: Supplementary file 1 [file plants-11-02975-s001.zip › plants-2009327-supplementary.pdf]

**Table S1:** The coefficient of determination ($R^2$) values and statistical significances (based on the F test) for the linear regressions between the measured individual plant parameters for wheat cultivar Káplár in two years. The regressions were established for individual plants (n = 60). $C_R$*: saturation root electrical capacitance; PH: plant height; CSA: stem cross-sectional area; SL: spike length; FLL: flag leaf length; FLW: flag leaf width; FLA: flag leaf area; SPAD: chlorophyll content in flag leaf; TAB: total aboveground biomass; GM: grain mass; GN: grain number. *$p < 0.05$, **$p < 0.01$, ***$p < 0.001$, NS not significant.

| Year | Parameter | $C_R$* | PH | CSA | PH×CSA | SL | FLL | FLW | FLA | SPAD | FLA×SPAD | TAB | GM |
|---|---|---|---|---|---|---|---|---|---|---|---|---|---|
| **2021** | **PH** | 0.281*** | | | | | | | | | | | |
| | **CSA** | 0.497*** | 0.424*** | | | | | | | | | | |
| | **PH×CSA** | 0.501*** | 0.681*** | 0.926*** | | | | | | | | | |
| | **SL** | 0.564*** | 0.487*** | 0.571*** | 0.630*** | | | | | | | | |
| | **FLL** | 0.594*** | 0.170** | 0.317*** | 0.315*** | 0.427*** | | | | | | | |
| | **FLW** | 0.621*** | 0.390*** | 0.741*** | 0.730*** | 0.607*** | 0.559*** | | | | | | |
| | **FLA** | 0.693*** | 0.293*** | 0.551*** | 0.549*** | 0.563*** | 0.886*** | 0.851*** | | | | | |
| | **SPAD** | 0.502*** | 0.465*** | 0.565*** | 0.630*** | 0.432*** | 0.375*** | 0.553*** | 0.499*** | | | | |
| | **FLA×SPAD** | 0.723*** | 0.390*** | 0.626*** | 0.655*** | 0.586*** | 0.804*** | 0.862*** | 0.955*** | 0.690*** | | | |
| | **TAB** | 0.524*** | 0.474*** | 0.481*** | 0.567*** | 0.512*** | 0.512*** | 0.591*** | 0.635*** | 0.443*** | 0.664*** | | |
| | **GM** | 0.560*** | 0.431*** | 0.436*** | 0.511*** | 0.510*** | 0.545*** | 0.558*** | 0.636*** | 0.427*** | 0.651*** | 0.945*** | |
| | **GN** | 0.489*** | 0.421*** | 0.400*** | 0.476*** | 0.495*** | 0.515*** | 0.527*** | 0.600*** | 0.399*** | 0.610*** | 0.922*** | 0.968*** |
| **2022** | **PH** | 0.249*** | | | | | | | | | | | |
| | **CSA** | 0.464*** | 0.354*** | | | | | | | | | | |
| | **PH×CSA** | 0.474*** | 0.564*** | 0.950*** | | | | | | | | | |
| | **SL** | 0.458*** | 0.482*** | 0.518*** | 0.571*** | | | | | | | | |
| | **FLL** | 0.474*** | 0.196*** | 0.395*** | 0.385*** | 0.485*** | | | | | | | |
| | **FLW** | 0.552*** | 0.318*** | 0.572*** | 0.574*** | 0.501*** | 0.612*** | | | | | | |
| | **FLA** | 0.564*** | 0.247*** | 0.509*** | 0.499*** | 0.518*** | 0.922*** | 0.841*** | | | | | |
| | **SPAD** | 0.628*** | 0.388*** | 0.571*** | 0.597*** | 0.538*** | 0.420*** | 0.620*** | 0.531*** | | | | |
| | **FLA×SPAD** | 0.665*** | 0.307*** | 0.598*** | 0.599*** | 0.559*** | 0.829*** | 0.852*** | 0.944*** | 0.732*** | | | |
| | **TAB** | 0.522*** | 0.210*** | 0.373*** | 0.375*** | 0.609*** | 0.499*** | 0.506*** | 0.563*** | 0.464*** | 0.603*** | | |
| | **GM** | 0.612*** | 0.268*** | 0.418*** | 0.436*** | 0.632*** | 0.520*** | 0.535*** | 0.591*** | 0.500*** | 0.641*** | 0.914*** | |
| | **GN** | 0.552*** | 0.302*** | 0.364*** | 0.399*** | 0.649*** | 0.521*** | 0.511*** | 0.568*** | 0.527*** | 0.620*** | 0.909*** | 0.906*** |

**Table S2:** The coefficient of determination ($R^2$) values and statistical significances (based on the F test) for the linear regressions between the measured individual plant parameters for wheat cultivar Kolo in two years. The regressions were established for individual plants (n = 60). For the abbreviations and symbols, see Table S1.

| Year | Parameter | $C_R$* | PH | CSA | PH×CSA | SL | FLL | FLW | FLA | SPAD | FLA×SPAD | TAB | GM |
|---|---|---|---|---|---|---|---|---|---|---|---|---|---|
| **2021** | **PH** | 0.275*** | | | | | | | | | | | |
| | **CSA** | 0.368*** | $0.051^{NS}$ | | | | | | | | | | |
| | **PH×CSA** | 0.511*** | 0.415*** | 0.787*** | | | | | | | | | |
| | **SL** | 0.338*** | 0.299*** | 0.307*** | 0.481*** | | | | | | | | |
| | **FLL** | 0.680*** | 0.213*** | 0.382*** | 0.498*** | 0.492*** | | | | | | | |
| | **FLW** | 0.507*** | $0.056^{NS}$ | 0.452*** | 0.400*** | 0.161** | 0.447*** | | | | | | |
| | **FLA** | 0.723*** | 0.163** | 0.470*** | 0.530*** | 0.395*** | 0.887*** | 0.764*** | | | | | |
| | **SPAD** | 0.424*** | 0.278*** | 0.352*** | 0.508*** | 0.515*** | 0.563*** | 0.265*** | 0.508*** | | | | |
| | **FLA×SPAD** | 0.688*** | 0.229*** | 0.473*** | 0.589*** | 0.491*** | 0.875*** | 0.626*** | 0.925*** | 0.752*** | | | |
| | **TAB** | 0.545*** | 0.239*** | 0.222*** | 0.362*** | 0.274*** | 0.468*** | 0.246*** | 0.455*** | 0.349*** | 0.500*** | | |
| | **GM** | 0.552*** | 0.256*** | 0.169** | 0.320*** | 0.284*** | 0.456*** | 0.245*** | 0.452*** | 0.343*** | 0.498*** | 0.908*** | |
| | **GN** | 0.508*** | 0.295*** | 0.193*** | 0.368*** | 0.359*** | 0.485*** | 0.219*** | 0.451*** | 0.397*** | 0.521*** | 0.866*** | 0.923*** |
| **2022** | **PH** | $0.043^{NS}$ | | | | | | | | | | | |
| | **CSA** | 0.216*** | 0.351*** | | | | | | | | | | |
| | **PH×CSA** | 0.173*** | 0.632*** | 0.915*** | | | | | | | | | |
| | **SL** | 0.262*** | 0.482*** | 0.618*** | 0.686*** | | | | | | | | |
| | **FLL** | 0.602*** | 0.083* | 0.144** | 0.305*** | 0.244*** | | | | | | | |
| | **FLW** | 0.581*** | 0.143** | 0.371*** | 0.338*** | 0.407*** | 0.619*** | | | | | | |
| | **FLA** | 0.681*** | 0.200*** | 0.241*** | 0.183*** | 0.330*** | 0.940*** | 0.823*** | | | | | |
| | **SPAD** | 0.328*** | 0.289*** | 0.409*** | 0.438*** | 0.465*** | 0.418*** | 0.579*** | 0.501*** | | | | |
| | **FLA×SPAD** | 0.662*** | 0.313*** | 0.333*** | 0.285*** | 0.422*** | 0.868*** | 0.834*** | 0.952*** | 0.699*** | | | |
| | **TAB** | 0.583*** | 0.257*** | 0.524*** | 0.510*** | 0.685*** | 0.380*** | 0.526*** | 0.489*** | 0.453*** | 0.559*** | | |
| | **GM** | 0.702*** | 0.179*** | 0.420*** | 0.396*** | 0.527*** | 0.399*** | 0.519*** | 0.504*** | 0.406*** | 0.558*** | 0.907*** | |
| | **GN** | 0.696*** | 0.186*** | 0.412*** | 0.395*** | 0.520*** | 0.403*** | 0.538*** | 0.513*** | 0.422*** | 0.570*** | 0.874*** | 0.956*** |

**Table S3:** The coefficient of determination ($R^2$) values and statistical significances (based on the F test) for the linear regressions between the measured individual plant parameters for wheat cultivar Lucilla in two years. The regressions were established for individual plants (n = 60). For the abbreviations and symbols, see Table S1.

| Year | Parameter | $C_R$* | PH | CSA | PH×CSA | SL | FLL | FLW | FLA | SPAD | FLA×SPAD | TAB | GM |
|---|---|---|---|---|---|---|---|---|---|---|---|---|---|
| **2021** | **PH** | 0.412*** | | | | | | | | | | | |
| | **CSA** | 0.405*** | 0.499*** | | | | | | | | | | |
| | **PH×CSA** | 0.475*** | 0.704*** | 0.945*** | | | | | | | | | |
| | **SL** | 0.451*** | 0.613*** | 0.758*** | 0.811*** | | | | | | | | |
| | **FLL** | 0.522*** | 0.342*** | 0.483*** | 0.492*** | 0.465*** | | | | | | | |
| | **FLW** | 0.650*** | 0.572*** | 0.576*** | 0.645*** | 0.667*** | 0.609*** | | | | | | |
| | **FLA** | 0.680*** | 0.482*** | 0.569*** | 0.617*** | 0.616*** | 0.862*** | 0.896*** | | | | | |
| | **SPAD** | 0.565*** | 0.537*** | 0.585*** | 0.637*** | 0.641*** | 0.611*** | 0.788*** | 0.757*** | | | | |
| | **FLA×SPAD** | 0.697*** | 0.493*** | 0.568*** | 0.627*** | 0.629*** | 0.794*** | 0.895*** | 0.973*** | 0.838*** | | | |
| | **TAB** | 0.645*** | 0.658*** | 0.665*** | 0.775*** | 0.790*** | 0.598*** | 0.665*** | 0.711*** | 0.605*** | 0.707*** | | |
| | **GM** | 0.701*** | 0.584*** | 0.611*** | 0.708*** | 0.788*** | 0.596*** | 0.673*** | 0.732*** | 0.585*** | 0.738*** | 0.906*** | |
| | **GN** | 0.662*** | 0.574*** | 0.620*** | 0.713*** | 0.799*** | 0.602*** | 0.664*** | 0.723*** | 0.586*** | 0.725*** | 0.912*** | 0.974*** |
| **2022** | **PH** | 0.149** | | | | | | | | | | | |
| | **CSA** | 0.193*** | 0.072* | | | | | | | | | | |
| | **PH×CSA** | 0.255*** | 0.308*** | 0.898*** | | | | | | | | | |
| | **SL** | 0.588*** | 0.471*** | 0.291*** | 0.481*** | | | | | | | | |
| | **FLL** | 0.611*** | 0.167** | 0.229*** | 0.219*** | 0.443*** | | | | | | | |
| | **FLW** | 0.558*** | 0.174*** | 0.288*** | 0.365*** | 0.643*** | 0.595*** | | | | | | |
| | **FLA** | 0.660*** | 0.277*** | 0.271*** | 0.294*** | 0.579*** | 0.923*** | 0.831*** | | | | | |
| | **SPAD** | 0.605*** | 0.384*** | 0.320*** | 0.474*** | 0.727*** | 0.468*** | 0.615*** | 0.573*** | | | | |
| | **FLA×SPAD** | 0.711*** | 0.155** | 0.304*** | 0.369*** | 0.682*** | 0.849*** | 0.839*** | 0.956*** | 0.750*** | | | |
| | **TAB** | 0.565*** | 0.203*** | 0.131** | 0.215*** | 0.748*** | 0.473*** | 0.484*** | 0.535*** | 0.486*** | 0.575*** | | |
| | **GM** | 0.608*** | 0.194*** | 0.212*** | 0.300*** | 0.776*** | 0.542*** | 0.580*** | 0.624*** | 0.548*** | 0.664*** | 0.896*** | |
| | **GN** | 0.576*** | 0.242*** | 0.139** | 0.235*** | 0.766*** | 0.490*** | 0.523*** | 0.569*** | 0.538*** | 0.624*** | 0.911*** | 0.920*** |

**Table S4:** The coefficient of determination ($R^2$) values and statistical significances (based on the F test) for the linear regressions between the measured individual plant parameters for wheat cultivar Ménrót in two years. The regressions were established for individual plants (n = 60). For the abbreviations and symbols, see Table S1.

| Year | Parameter | $C_R$* | PH | CSA | PH×CSA | SL | FLL | FLW | FLA | SPAD | FLA×SPAD | TAB | GM |
|---|---|---|---|---|---|---|---|---|---|---|---|---|---|
| **2021** | **PH** | 0.165** | | | | | | | | | | | |
| | **CSA** | 0.355*** | 0.357*** | | | | | | | | | | |
| | **PH×CSA** | 0.354*** | 0.597*** | 0.939*** | | | | | | | | | |
| | **SL** | 0.385*** | 0.387*** | 0.524*** | 0.579*** | | | | | | | | |
| | **FLL** | 0.533*** | 0.081* | 0.354*** | 0.313*** | 0.563*** | | | | | | | |
| | **FLW** | 0.595*** | 0.267*** | 0.650*** | 0.629*** | 0.598*** | 0.650*** | | | | | | |
| | **FLA** | 0.604*** | 0.152** | 0.500*** | 0.462*** | 0.623*** | 0.947*** | 0.840*** | | | | | |
| | **SPAD** | 0.533*** | 0.343*** | 0.474*** | 0.518*** | 0.539*** | 0.398*** | 0.563*** | 0.500*** | | | | |
| | **FLA×SPAD** | 0.654*** | 0.221*** | 0.551*** | 0.537*** | 0.669*** | 0.868*** | 0.842*** | 0.952*** | 0.702*** | | | |
| | **TAB** | 0.633*** | 0.206*** | 0.447*** | 0.450*** | 0.474*** | 0.456*** | 0.524*** | 0.524*** | 0.512*** | 0.581*** | | |
| | **GM** | 0.696*** | 0.196*** | 0.437*** | 0.435*** | 0.437*** | 0.420*** | 0.558*** | 0.510*** | 0.518*** | 0.571*** | 0.887*** | |
| | **GN** | 0.628*** | 0.219*** | 0.390*** | 0.409*** | 0.437*** | 0.389*** | 0.508*** | 0.470*** | 0.446*** | 0.516*** | 0.886*** | 0.960*** |
| **2022** | **PH** | 0.155** | | | | | | | | | | | |
| | **CSA** | 0.291*** | 0.005$^{NS}$ | | | | | | | | | | |
| | **PH×CSA** | 0.402*** | 0.154** | 0.889*** | | | | | | | | | |
| | **SL** | 0.415*** | 0.149** | 0.326*** | 0.185*** | | | | | | | | |
| | **FLL** | 0.621*** | 0.214*** | 0.177*** | 0.205*** | 0.200*** | | | | | | | |
| | **FLW** | 0.507*** | 0.195*** | 0.122** | 0.145** | 0.194*** | 0.684*** | | | | | | |
| | **FLA** | 0.625*** | 0.210*** | 0.166** | 0.194*** | 0.225*** | 0.954*** | 0.852*** | | | | | |
| | **SPAD** | 0.599*** | 0.209*** | 0.145** | 0.256*** | 0.271*** | 0.438*** | 0.353*** | 0.441*** | | | | |
| | **FLA×SPAD** | 0.719*** | 0.303*** | 0.183*** | 0.243*** | 0.283*** | 0.907*** | 0.772*** | 0.940*** | 0.668*** | | | |
| | **TAB** | 0.620*** | 0.176*** | 0.219*** | 0.286*** | 0.281*** | 0.340*** | 0.292*** | 0.347*** | 0.295*** | 0.390*** | | |
| | **GM** | 0.697*** | 0.115** | 0.225*** | 0.312*** | 0.329*** | 0.398*** | 0.338*** | 0.407*** | 0.351*** | 0.458*** | 0.905*** | |
| | **GN** | 0.696*** | 0.131** | 0.246*** | 0.345*** | 0.346*** | 0.397*** | 0.331*** | 0.401*** | 0.355*** | 0.453*** | 0.889*** | 0.941*** |

**Table S5:** The coefficient of determination ($R^2$) values and statistical significances (based on the F test) for the linear regressions between the measured individual plant parameters for wheat cultivar Pántlika in two years. The regressions were established for individual plants (n = 60). For the abbreviations and symbols, see Table S1.

| Year | Parameter | $C_R$* | PH | CSA | PH×CSA | SL | FLL | FLW | FLA | SPAD | FLA×SPAD | TAB | GM |
|---|---|---|---|---|---|---|---|---|---|---|---|---|---|
| **2021** | **PH** | 0.361*** | | | | | | | | | | | |
| | **CSA** | 0.533*** | 0.580*** | | | | | | | | | | |
| | **PH×CSA** | 0.533*** | 0.821*** | 0.921*** | | | | | | | | | |
| | **SL** | 0.572*** | 0.620*** | 0.744*** | 0.778*** | | | | | | | | |
| | **FLL** | 0.600*** | 0.212*** | 0.546*** | 0.447*** | 0.584*** | | | | | | | |
| | **FLW** | 0.664*** | 0.466*** | 0.707*** | 0.683*** | 0.733*** | 0.733*** | | | | | | |
| | **FLA** | 0.688*** | 0.347*** | 0.668*** | 0.602*** | 0.700*** | 0.935*** | 0.910*** | | | | | |
| | **SPAD** | 0.626*** | 0.655*** | 0.638*** | 0.736*** | 0.729*** | 0.478*** | 0.636*** | 0.592*** | | | | |
| | **FLA×SPAD** | 0.738*** | 0.535*** | 0.731*** | 0.750*** | 0.788*** | 0.800*** | 0.871*** | 0.913*** | 0.827*** | | | |
| | **TAB** | 0.551*** | 0.612*** | 0.565*** | 0.676*** | 0.739*** | 0.446*** | 0.605*** | 0.572*** | 0.750*** | 0.741*** | | |
| | **GM** | 0.560*** | 0.620*** | 0.630*** | 0.726*** | 0.758*** | 0.495*** | 0.658*** | 0.629*** | 0.754*** | 0.794*** | 0.909*** | |
| | **GN** | 0.548*** | 0.606*** | 0.614*** | 0.710*** | 0.762*** | 0.507*** | 0.629*** | 0.630*** | 0.748*** | 0.793*** | 0.895*** | 0.983*** |
| **2022** | **PH** | 0.268*** | | | | | | | | | | | |
| | **CSA** | 0.129** | 0.244*** | | | | | | | | | | |
| | **PH×CSA** | 0.225*** | 0.538*** | 0.905*** | | | | | | | | | |
| | **SL** | 0.381*** | 0.347*** | 0.257*** | 0.170** | | | | | | | | |
| | **FLL** | 0.484*** | 0.290*** | 0.265*** | 0.161** | 0.492*** | | | | | | | |
| | **FLW** | 0.634*** | 0.388*** | 0.290*** | 0.207*** | 0.580*** | 0.674*** | | | | | | |
| | **FLA** | 0.601*** | 0.366*** | 0.302*** | 0.210*** | 0.573*** | 0.930*** | 0.878*** | | | | | |
| | **SPAD** | 0.509*** | 0.319*** | 0.370*** | 0.234*** | 0.417*** | 0.389*** | 0.437*** | 0.428*** | | | | |
| | **FLA×SPAD** | 0.682*** | 0.427*** | 0.377*** | 0.284*** | 0.598*** | 0.848*** | 0.832*** | 0.926*** | 0.676*** | | | |
| | **TAB** | 0.557*** | 0.271*** | 0.274*** | 0.437*** | 0.312*** | 0.430*** | 0.367*** | 0.442*** | 0.460*** | 0.533*** | | |
| | **GM** | 0.609*** | 0.327*** | 0.254*** | 0.368*** | 0.340*** | 0.427*** | 0.413*** | 0.468*** | 0.470*** | 0.566*** | 0.918*** | |
| | **GN** | 0.603*** | 0.192*** | 0.242*** | 0.351*** | 0.368*** | 0.399*** | 0.418*** | 0.450*** | 0.412*** | 0.521*** | 0.872*** | 0.936*** |

**Table S6:** The slope (±SE) and y-intercept (±SE) for the linear regressions between the saturation root electrical capacitance ($C_R$*) and various plant parameters investigated in five wheat cultivars in two years. The regressions were established for individual plants (n = 60). For the abbreviations, see Table S1.

| Year | Parameter | Káplár | | Kolo | | Lucilla | | Ménrót | | Pántlika | |
|---|---|---|---|---|---|---|---|---|---|---|---|
| | | Slope | Intercept | Slope | Intercept | Slope | Intercept | Slope | Intercept | Slope | Intercept |
| 2021 | PH | 0.079±0.017 | 2.73±1.04 | 0.097±0.021 | 1.68±1.43 | 0.101±0.016 | 1.34±1.16 | 0.085±0.025 | 3.08±1.70 | 0.104±0.018 | 1.74±1.11 |
| | CSA | 0.366±0.048 | 4.44±0.43 | 0.426±0.073 | 3.94±0.77 | 0.322±0.051 | 5.27±0.56 | 0.314±0.055 | 5.55±0.59 | 0.495±0.061 | 3.48±0.58 |
| | PH×CSA | 0.424±0.055 | 5.33±0.32 | 0.585±0.074 | 4.16±0.55 | 0.353±0.049 | 5.91±0.41 | 0.367±0.065 | 6.21±0.48 | 0.525±0.065 | 5.05±0.39 |
| | SL | 0.778±0.090 | 2.27±0.63 | 0.969±0.178 | 1.86±1.20 | 0.787±0.114 | 3.38±0.78 | 0.911±0.151 | 3.00±0.97 | 1.043±0.119 | 1.43±0.76 |
| | FLL | 0.353±0.038 | 3.34±0.48 | 0.492±0.044 | 1.70±0.61 | 0.567±0.071 | 1.58±0.90 | 0.470±0.058 | 3.31±0.69 | 0.502±0.054 | 0.94±0.77 |
| | FLW | 0.528±0.054 | 2.14±0.57 | 0.701±0.061 | 0.28±1.13 | 0.553±0.053 | 1.81±0.67 | 0.797±0.086 | 0.71±1.038 | 0.859±0.080 | 1.35±0.88 |
| | FLA | 0.279±0.024 | 4.94±0.25 | 0.362±0.029 | 3.78±0.38 | 0.330±0.030 | 4.76±0.37 | 0.364±0.038 | 4.95±0.42 | 0.380±0.033 | 3.56±0.40 |
| | SPAD | 0.200±0.026 | 1.68±0.78 | 0.223±0.034 | 1.29±1.09 | 0.211±0.024 | 1.52±0.83 | 0.271±0.033 | 0.41±1.14 | 0.246±0.025 | 0.99±0.72 |
| | FLA×SPAD | .0071±.0005 | 5.54±0.18 | .0078±.0007 | 5.17±0.30 | .0064±.0006 | 5.97±0.26 | .0084±.0008 | 5.71±0.31 | .0088±.0006 | 4.98±0.26 |
| | TAB | 1.054±0.131 | 5.82±0.24 | 2.051±0.244 | 3.95±0.53 | 1.987±0.194 | 4.66±0.41 | 1.695±0.170 | 5.17±0.38 | 1.735±0.206 | 4.82±0.40 |
| | GM | 2.119±0.247 | 5.90±0.22 | 4.855±0.574 | 4.15±0.51 | 4.168±0.357 | 5.20±0.32 | 3.558±0.309 | 5.83±0.27 | 3.646±0.424 | 5.48±0.32 |
| | GN | 0.084±0.011 | 5.88±0.25 | 0.177±0.023 | 4.63±0.50 | 0.188±0.017 | 4.95±037 | 0.152±0.015 | 5.83±0.31 | 0.177±0.021 | 5.33±0.34 |
| 2022 | PH | 0.095±0.022 | 2.03±1.53 | 0.033±0.021 | 6.35±1.56 | 0.088±0.027 | 3.82±1.88 | 0.153±0.047 | 0.64±3.63 | 0.146±0.032 | 0.38±2.16 |
| | CSA | 0.305±0.043 | 5.41±0.48 | 0.248±0.073 | 6.30±0.65 | 0.225±0.060 | 7.134±0.73 | 0.388±0.080 | 5.67±1.14 | 0.258±0.088 | 7.34±0.1.01 |
| | PH×CSA | 0.352±0.049 | 5.98±0.32 | 0.223±0.064 | 7.11±0.52 | 0.331±0.074 | 7.11±0.62 | 0.547±0.088 | 5.18±0.98 | 0.380±0.093 | 7.32±0.74 |
| | SL | 0.864±0.123 | 2.56±0.88 | 0.716±0.158 | 4.11±1.05 | 1.049±0.115 | 2.38±0.82 | 1.009±0.157 | 3.60±1.19 | 1.020±0.171 | 2.82±1.26 |
| | FLL | 0.404±0.056 | 3.53±0.73 | 0.392±0.042 | 3.57±0.57 | 0.515±0.054 | 2.73±0.75 | 0.489±0.050 | 3.38±0.77 | 0.444±0.060 | 3.00±0.99 |
| | FLW | 0.631±0.075 | 0.91±0.93 | 0.685±0.076 | 0.25±0.96 | 0.717±0.084 | 0.053±1.16 | 0.777±0.098 | 0.680±1.37 | 0.930±0.093 | 1.36±1.16 |
| | FLA | 0.291±0.033 | 5.20±0.42 | 0.285±0.026 | 5.18±0.34 | 0.322±0.030 | 5.20±0.45 | 0.303±0.031 | 6.33±0.52 | 0.319±0.034 | 5.13±0.54 |
| | SPAD | 0.210±0.021 | 1.40±0.74 | 0.166±0.032 | 3.51±1.01 | 0.255±0.027 | 0.487±0.991 | 0.294±0.032 | 0.40±1.25 | 0.266±0.034 | 1.66±1.19 |
| | FLA×SPAD | .0065±.0006 | 5.92±0.27 | .0063±.0006 | 6.17±0.26 | .0065±.0005 | 6.35±0.30 | .0063±.0005 | 7.13±0.35 | .0075±.0007 | 6.41±0.36 |
| | TAB | 1.390±0.175 | 5.33±0.44 | 0.971±0.108 | 6.61±0.27 | 1.147±0.169 | 6.25±0.42 | 1.749±0.180 | 6.11±0.54 | 1.685±0.197 | 6.28±0.48 |
| | GM | 2.923±0.306 | 5.29±0.37 | 2.140±0.183 | 6.69±0.20 | 3.076±0.324 | 6.476±0.366 | 3.532±0.306 | 6.42±0.43 | 3.908±0.411 | 6.21±0.44 |
| | GN | 0.124±0.015 | 5.06±0.44 | 0.101±0.009 | 6.48±0.22 | 0.140±0.016 | 6.28±0.41 | 0.165±0.014 | 5.97±0.47 | 0.181±0.019 | 6.02±0.47 |
